# Virtual reality as a non-pharmacological intervention for pediatric preoperative anxiety: A quality improvement initiative and retrospective analysis

**DOI:** 10.1371/journal.pdig.0001219

**Published:** 2026-11-05

**Authors:** Walaa S. Abu Rukbah, Narendra Vadlamudi, Siri Garrett, Suad Alshammari, Dayanjan S. Wijesinghe

**Affiliations:** 1 Department of Pharmacy Practice, Faculty of Pharmacy, University of Tabuk, Tabuk, Saudi Arabia; 2 Children’s Hospital of Richmond at Virginia Commonwealth University, Richmond, Virginia, United States of America; 3 Department of Clinical Practice, College of Pharmacy, Northern Border University, Rafha, Saudi Arabia; 4 Department of Pharmacotherapy and Outcomes Science, School of Pharmacy, Virginia Commonwealth University, Richmond, Virginia, United States of America; Yonsei University College of Medicine, KOREA, REPUBLIC OF

## Abstract

Preoperative anxiety affects a majority of children undergoing surgery and is associated with adverse perioperative and psychological outcomes. Virtual reality (VR) is a promising non-pharmacological option, but real-world implementation data remain limited. We report a nine-month quality-improvement (QI) initiative that implemented a custom VR intervention in a pediatric preoperative holding area, followed by retrospective analysis of prospectively collected data. Using a single-arm, pre-post design without a comparison group, we evaluated within-subject changes in self-reported distress and fear, physiological indicators, and stakeholder experience in 80 children aged 6–15 years. Between the pre-VR and immediately post-VR assessments, distress (0–10 Numerical Rating Scale) decreased by a median of 2.0 points (median 3.0 to 1.0; p < 0.001) and fear (0–4 Children's Fear Scale) by a median of 1.0 point (1.0 to 0.0; p < 0.001). Heart rate fell by a mean of 5.8 beats/min and systolic blood pressure by 4.1 mmHg (both p < 0.001). Acceptance was high: 91% of children were willing to use VR again, 95% of parents would recommend it, and all participating nurses endorsed it, with few, minor adverse effects (3 of 80). In this uncontrolled QI initiative, VR was feasible and safe to implement and was accompanied by clinically meaningful within-subject reductions in anxiety and physiological arousal. Because the design included no comparison group, these changes cannot be attributed to VR alone and may partly reflect natural settling, acclimation, non-specific attention, or regression to the mean. Controlled trials are needed to establish comparative effectiveness before routine adoption.

## Author summary

Many children feel anxious before surgery, and this anxiety can make surgery harder for children and families. Calming medicines can help but may cause side effects, so we wanted to see whether virtual reality (VR) could offer a drug-free alternative. Our team built a calm, child-friendly virtual world and, over nine months, made it part of routine care in the area where children wait before surgery at a children’s hospital in Richmond, Virginia. We kept improving the program based on feedback from children, parents, and nurses. Eighty children aged 6–15 took part. After using VR, children reported feeling less distressed and less afraid than before, and their heart rate and blood pressure were lower. Nearly all children said they would use VR again, almost all parents would recommend it, and nurses found it easy to fit into their work. Side effects were rare and mild. Because we had no comparison group, controlled trials are still needed to confirm how much of this improvement comes from VR itself. Our experience shows that VR can be built into everyday surgical care safely and smoothly, and that it offers a promising, drug-free way to support children facing surgery.

## Introduction

Preoperative anxiety is among the most prevalent and clinically significant challenges in pediatric surgical care, affecting 50–75% of children undergoing surgical procedures [1,2]. This anxiety manifests through psychological distress, physiological stress responses, and behavioral difficulties that can compromise both immediate perioperative safety and long-term psychological outcomes [3,4]. Children experiencing high preoperative anxiety demonstrate increased risk of difficult anesthesia induction, emergence delirium, postoperative pain, prolonged recovery times, and development of negative healthcare attitudes that persist into subsequent medical encounters [1].

Current approaches to managing pediatric preoperative anxiety include pharmacological premedication, behavioral preparation programs, and parental presence during induction. While oral midazolam remains the most commonly employed anxiolytic, it carries risks of paradoxical reactions, respiratory depression, and delayed discharge [2,3,5]. Non-pharmacological interventions such as therapeutic play, music therapy, and clown therapy have demonstrated anxiety-reducing effects but require specialized personnel and may not be universally available or acceptable to all children [2,6]. These limitations have driven investigation of novel technological interventions that can provide consistent, accessible, and effective anxiety management without pharmacological side effects.

Virtual reality has emerged as a promising non-pharmacological intervention for procedural anxiety and pain management across diverse medical contexts. The theoretical foundation for virtual reality's anxiolytic effects derives from attention-based distraction models, which posit that immersive virtual environments engage cognitive and sensory resources sufficiently to reduce processing of anxiety-provoking stimuli [4,7]. By creating compelling alternative realities that capture visual, auditory, and proprioceptive attention, virtual reality may interrupt the anxiety response cycle and reduce both subjective distress and objective physiological arousal.

Despite growing interest in pediatric virtual reality, significant knowledge gaps persist regarding real-world implementation. Most existing studies have examined virtual reality in controlled research settings with brief procedural applications, leaving questions about feasibility, stakeholder acceptance, and sustainable integration into routine clinical workflows [1,3,8,9]. Furthermore, few investigations have employed comprehensive outcome assessment encompassing psychological, physiological, and experiential measures across multiple stakeholder groups including patients, families, and healthcare providers.

To address these gaps, we conducted a nine-month quality improvement initiative implementing custom-designed virtual reality interventions for pediatric preoperative anxiety management, followed by retrospective analysis of prospectively collected implementation data. Our study objectives were to: (1) evaluate changes in pediatric preoperative distress, fear, and physiological markers following virtual reality intervention; (2) identify predictors of clinically significant anxiety reduction; (3) assess stakeholder experience including patient satisfaction, parent anxiety, and healthcare provider acceptance; and (4) assess overall implementation success across clinical response and stakeholder experience, with baseline predictors of individual outcomes explored in Supporting Information.

## Materials and methods

### Ethics statement

The Virginia Commonwealth University Institutional Review Board determined that this study, a retrospective analysis of de-identified data originally collected during a clinical quality improvement initiative, is not research involving human subjects and that its review and approval were not required (VCU IRB Protocol #HM20032312; ClinicalTrials.gov registration NCT07280910). Written informed consent was obtained from the parent/guardian of each participant under 18 years of age. The quality improvement project was conducted under standard hospital operational protocols at VCU Children's Hospital. During the quality improvement implementation phase, written informed consent was obtained from parents or legal guardians and age-appropriate assent from pediatric participants prior to virtual reality intervention, following detailed explanation of procedures, potential risks, benefits, and participant rights. All consent documentation was written at an eighth grade reading level to ensure comprehension across diverse literacy levels. The study was conducted in accordance with the Declaration of Helsinki and International Conference on Harmonisation Good Clinical Practice guidelines.

### Study design and setting

We conducted a retrospective analysis of prospectively collected data generated during a nine-month quality improvement initiative (October 2023 through June 2024) implementing virtual reality interventions for pediatric preoperative anxiety management. The initiative took place at VCU Children's Hospital in Richmond, Virginia, specifically within the pediatric preoperative holding area, a 16-bed unit managing approximately 1,000 surgical cases annually. The study employed a single-arm, pre-post design to examine within-subject changes in psychological and physiological outcomes associated with virtual reality intervention, while characterizing stakeholder experience and testing predictors of clinically meaningful improvement under routine clinical conditions. Consistent with its quality-improvement purpose, the initiative was designed to evaluate and iteratively improve real-world care rather than to test a hypothesis under experimental control, and was classified by the Institutional Review Board as non-human-subjects research (see Ethics Statement). Because the design included no concurrent control group, no formal causal inference was undertaken; all analyses characterize within-subject change and association rather than the causal effect of virtual reality. The initiative followed a mixed-methods structure in which quantitative pre-post and stakeholder-rating data were complemented by qualitative feedback gathered across successive Plan-Do-Study-Act cycles, with the qualitative findings used to refine the intervention and to contextualize, rather than to confirm, the quantitative results.

### Quality improvement framework

The implementation followed Plan-Do-Study-Act methodology across five complete cycles, each lasting 4–6 weeks. This iterative approach enabled continuous refinement of virtual reality intervention protocols based on real-time data collection and stakeholder feedback. Each cycle followed structured processes of planning interventions based on previous findings, implementing changes in clinical practice, studying outcomes through systematic data collection and analysis, and acting on results to standardize successful modifications. The initiative aimed to: (1) implement virtual reality technology as non-pharmacological intervention for pediatric preoperative anxiety; (2) optimize clinical workflow integration; (3) maximize satisfaction among patients, parents, and healthcare providers; and (4) continuously refine protocols based on stakeholder feedback and outcomes.

Cycle 1 (October-November 2023) established feasibility and safety through standardized 3–5 minute intervention sessions with systematic documentation of adverse events, setup time, technical failures, and age-stratified usability assessment. Cycle 2 (January-February 2024) introduced tablet-based parental viewing capability while establishing baseline effectiveness metrics with standardized five-minute sessions. Cycle 3 (March-April 2024) implemented flexible session duration (5–20 minutes) based on patient preference and expanded clinical applications beyond procedural support to general preoperative anxiety management. Cycle 4 (May 2024) validated the refined protocol with minimum 10-minute session duration and indication-specific implementation. Cycle 5 (June 2024) completed data collection while assessing operational sustainability and staff competency.

### Study population and eligibility

#### Pediatric participants.

Eligible patients were children aged 6–15 years scheduled for elective surgical procedures, capable of understanding instructions and providing self-report on validated anxiety scales, able to wear a virtual reality headset safely, and accompanied by a parent or legal guardian able to provide informed consent. Exclusion criteria included history of seizures or severe motion sickness, sensory or cognitive impairment preventing safe virtual reality use, active infection or open wounds on head or face, positive influenza-like illness screen on day of surgery, and any additional contraindication identified at clinician discretion.

#### Parent or caregiver participants.

Parents or legal guardians present during the preoperative period and willing to complete anxiety and satisfaction measures were eligible for participation.

#### Healthcare provider participants.

Preoperative nurses involved in virtual reality setup and supervision and willing to complete satisfaction and feasibility ratings were included.

Participants were recruited using convenience sampling during routine preoperative visits. The nursing team identified eligible patients from daily surgical schedules and notified the quality improvement team of potential participants. Initial recruitment occurred three days weekly (Tuesday, Wednesday, Friday), expanding to five days weekly with extended hours by Cycle 3 based on patient volume analysis.

### Virtual reality intervention

#### Technology selection.

The Meta Quest 2 virtual reality headset was selected following systematic evaluation of available technologies against criteria including pediatric safety profile, ease of disinfection between patients, age-appropriate content availability, cost-effectiveness, and technical support requirements. The device offered wireless functionality important for the clinical environment and met all safety and practical requirements.

#### Content development.

Custom virtual reality environments were developed over 10 months (November 2022 through August 2023) using Unity 3D software, incorporating evidence-based design principles for pediatric anxiety reduction. The environment featured an interactive children's room containing familiar toys and objects responsive to gaze and controller input, virtual television displaying age-appropriate content, warm color palette (predominantly blues and greens) associated with reduced anxiety, spatial audio with background music at 60–70 beats per minute, and consistent lighting to minimize visual fatigue. A secondary beach scene featured gentle ocean waves, natural elements including palm trees and cloud movements, and calming environmental sounds.

To minimize motion-related discomfort, the environment employed fixed viewpoint design where users remained in stable virtual locations with teleportation-based navigation allowing point-to-point movement between specific locations without experiencing visual acceleration. This approach minimized vestibular-visual conflict, a primary factor in motion-related discomfort particularly relevant to pediatric populations. Effectiveness of this design was confirmed through pilot testing with 15 pediatric patients, with zero reports of motion discomfort or nausea.

#### Intervention protocol.

Following quality improvement refinement cycles, the final protocol consisted of: (1) eligibility screening by Child Life Specialist; (2) informed consent and assent procedures; (3) baseline assessment including demographic information, physiological measures from bedside monitors, and self-reported distress and fear using validated scales; (4) virtual reality headset fitting and orientation; (5) patient-directed virtual reality session with minimum 10-minute duration and maximum 20-minute duration based on patient preference and clinical workflow; (6) concurrent parental viewing via tablet-based mirroring system; (7) immediate post-intervention assessment of distress, fear, and physiological parameters; and (8) satisfaction feedback from patients, parents, and nursing staff. The intervention evolved across the early Plan-Do-Study-Act cycles: from Cycle 4 onward the core delivery was stabilized to a minimum 10-minute, patient-directed session using the same custom environment, headset, and administration sequence, although the clinical indications for offering virtual reality continued to broaden and Cycle 5 emphasized sustainability rather than protocol change. Session duration and cycle were therefore not fully uniform across the full sample; duration was examined as a covariate in the predictor models (with no significant association with clinical response) and cross-cycle consistency was examined directly (see Statistical Analysis), but residual protocol evolution is acknowledged as a limitation rather than fully controlled.

### Outcome measures

#### Primary outcomes.

Procedural distress was measured using a 0–10 Numerical Rating Scale (NRS), where 0 indicated “no distress” and 10 indicated “extreme distress.” Although originally validated for pediatric pain, the NRS has also demonstrated validity for assessing distress and emotional responses during medical procedures. Fear was assessed using the Children’s Fear Scale (CFS), a 5-face pictorial measure scored 0–4, validated for children aged 7–12 years in procedural contexts. Both measures were administered at baseline (T1) and immediately post-intervention (T2) using standardized prompts. Although the Children's Fear Scale was originally validated in children aged 7–12 years, it was applied here across the full 6–15 year eligibility range; ratings for participants outside the original validation range should be interpreted with corresponding caution. Both instruments are brief, single-item measures selected for feasibility and low respondent burden in the perioperative setting; as simplified scales they may be less sensitive than multi-item anxiety inventories and are subject to floor effects, which we consider when interpreting the findings (see Limitations).

#### Secondary outcomes.

Physiological parameters included heart rate (beats/min) and systolic blood pressure (mmHg), recorded five minutes pre-intervention and immediately post-intervention using calibrated bedside monitors. Parent anxiety was measured using the 6-item State-Trait Anxiety Inventory short form (STAI-6), which provides a brief, validated measure of situational anxiety. Parents completed the STAI-6 at baseline and post-intervention.

#### Satisfaction and feasibility.

Satisfaction was assessed using 5-point Likert scales (1 = “extremely dissatisfied” to 5 = “extremely satisfied”). Children and parents independently rated intervention satisfaction and overall procedural experience. Nurses completed parallel scales evaluating feasibility, satisfaction, and perceived effectiveness in anxiety management. Satisfaction and experience ratings were collected by the study team immediately after each session using standardized forms; because these ratings were not fully anonymous and participants were aware that they were receiving a novel intervention, the satisfaction measures are susceptible to social-desirability and response bias and are interpreted with this limitation in mind (see Limitations).

### Data collection procedures

Patient recruitment was facilitated by the unit's Child Life Specialist who conducted initial eligibility screening and provided information to parents and patients. Eligible patients and accompanying parents were referred to the research team for enrollment. Research personnel explained the study, obtained written informed consent, and proceeded with baseline data collection.

Pre-intervention assessment included demographic information (age, gender), baseline physiological measures (heart rate, systolic blood pressure) recorded from bedside monitors, and participant self-reported baseline distress and fear assessed using validated scales. For parents consenting to participate, baseline state anxiety was measured using the State-Trait Anxiety Inventory-6.

During virtual reality intervention, process metrics were documented including session start and end times to calculate total duration, primary clinical indication for use (procedural support versus general anxiety), and any observed adverse effects or technical issues. Upon virtual reality completion, immediate post-intervention assessment was conducted involving readministration of distress and fear scales, post-intervention heart rate and blood pressure recorded from monitors, participant feedback via satisfaction scales, and indication of willingness to use virtual reality in future procedures. Participating parents simultaneously completed post-intervention anxiety measurements and provided feedback regarding their perceptions of intervention effectiveness. Nursing staff completed implementation feasibility and satisfaction assessments.

### Data management and quality assurance

All data were de-identified at the point of collection using participant codes and stored in a secure, password-protected database. Data entry was checked against original forms to ensure accuracy, and the dataset was reviewed for out-of-range values and logical inconsistencies. Research staff verified completeness of data forms at the time of collection. Regular audits and monitoring ensured >95% data completeness and integrity across all study variables.

### Statistical analysis

All analyses were performed in Python using the pandas, statsmodels, and SciPy libraries. Given the single-arm, pre-post design, analyses characterized within-subject change between the pre-VR (T1) and immediately post-VR (T2) assessments; in the absence of a concurrent control group, no between-group or causal comparisons were undertaken. Continuous variables are summarized as mean plus or minus standard deviation and ordinal or non-normally distributed variables as median with interquartile range. Distributional assumptions were examined using the Shapiro-Wilk test together with inspection of Q-Q plots. All four pre-post outcomes (distress and fear as the primary psychological outcomes, and heart rate and systolic blood pressure as secondary physiological outcomes) were complete for all 80 participants, so these analyses are complete-case analyses on the full sample; the small amount of missingness elsewhere in the record was confined to fields not used in the primary analyses and required no imputation.

Paired pre-post comparisons used the Wilcoxon signed-rank test for the ordinal distress (0–10 Numerical Rating Scale) and fear (0–4 Children's Fear Scale) outcomes and paired t-tests for the approximately normally distributed physiological outcomes (heart rate and systolic blood pressure). To limit inflation of the Type I error rate across the four pre-post outcome comparisons, we additionally applied Holm and Benjamini-Hochberg corrections as a sensitivity analysis; all four comparisons remained statistically significant after adjustment (Holm-adjusted p ranging from 3.3 x 10^-9 for distress to 1.7 x 10^-4 for heart rate). Confidence intervals for baseline and post-intervention medians were obtained by bootstrap resampling; confidence intervals for the paired median change in the ordinal distress and fear outcomes were obtained using the Hodges-Lehmann estimator corresponding to the Wilcoxon signed-rank test.

Clinically meaningful response was defined for each outcome, for the purposes of this retrospective analysis, as an absolute change: a reduction of at least 2 points on the 0–10 distress scale and of at least 1 point on the 0–4 fear scale. These operational thresholds were adopted during the revision and were not pre-registered. The 2-point distress threshold is supported by pediatric pain and distress studies in which the minimal clinically significant difference for self-report numerical and faces scales is approximately 2 points and is more stable across baseline levels than percentage-based change, which is mathematically baseline-dependent and undefined for the substantial subset of participants with a baseline score of zero [20–23]. The Children's Fear Scale has no established minimal clinically important difference; the 1-point threshold is therefore a reasoned extrapolation from this faces-scale literature and is interpreted with corresponding caution. Percentage-based thresholds (reductions of at least 30% and at least 50%) are additionally reported as labeled secondary and sensitivity analyses to preserve comparability with prior reports; no percentage threshold was applied to the 0–4 fear scale. Under the primary definition, 46 of 80 participants (57.5%) met the distress threshold and 44 of 80 (55.0%) met the fear threshold (Table 3).

Predictors of response were examined using multivariable logistic regression with three pre-specified covariates: age, baseline score, and virtual reality session duration. For comparability with the originally reported analyses, these predictor models retain the secondary percentage-based responder definition (a reduction of at least 50%; Table 5) rather than the absolute primary definition, and the fear model was fitted on the fear scale as originally recorded. This choice is deliberate and conservative: refitting the models on the absolute primary definition substantially inflates the baseline-score odds ratios (for example, from 1.80 to 7.72 for fear), because a fixed absolute reduction is unattainable for the 22 of 80 participants already at the fear-scale floor, so baseline score partly determines the outcome by construction. The percentage-based models are therefore the more conservative estimate of the baseline-response association, and both specifications remain subject to the regression-to-the-mean caution described in the Discussion. Model adequacy was assessed using the number of events per variable, which met the conventional minimum of 10 (for example, 11.3 in the distress-response model); nonetheless, given the modest sample size and the low pseudo-R-squared values, the regression models are interpreted as exploratory and hypothesis-generating rather than confirmatory. They were estimated by ordinary maximum likelihood, and their robustness was assessed through influence diagnostics and a bootstrap optimism-corrected assessment of discrimination rather than through a single robust-covariance estimator. Because baseline score was itself a predictor, and because participants with the highest baseline scores have the greatest scope for improvement, the baseline-as-predictor associations are susceptible to regression to the mean; we therefore additionally examined these relationships using analysis of covariance and baseline-stratified summaries and interpret them cautiously (see Discussion).

Because the intervention protocol was refined across five Plan-Do-Study-Act cycles, we examined whether the primary distress outcome was consistent across cycles using the Kruskal-Wallis test. The cycle index was not recorded per patient; it was reconstructed from enrollment order together with the cycle sizes reported in the quality-improvement report (10, 11, 13, 29, and 17 patients for cycles 1–5) and validated against independently reported per-cycle statistics. This test did not detect a significant between-cycle difference in distress change (Kruskal-Wallis p = 0.52); per-cycle medians for all outcomes are tabulated in the Supporting Information (S2 Table). Given the small per-cycle samples, this is a descriptive sensitivity check rather than evidence of a stable intervention effect across cycles, and the evolving, non-constant nature of the intervention across cycles is retained as an internal-validity limitation.

Open-ended parent feedback was analyzed qualitatively using an inductive thematic approach: free-text responses were grouped into recurring themes, each summarized by its prevalence and representative quotations (Table 9), and theme co-occurrence was tabulated. Because the qualitative component was embedded within a quality-improvement initiative, a formally pre-specified codebook and independent double-coding with a quantified inter-rater reliability statistic were not established; the thematic findings are accordingly interpreted as contextual and hypothesis-generating rather than confirmatory (see Limitations).

### Sample size determination

No a priori sample-size calculation was performed, consistent with the quality-improvement nature of the initiative; the analytic sample comprised all eligible pediatric patients enrolled during the nine-month implementation period (N = 80). The achieved sample size was evaluated post hoc for analytic adequacy: with 46 responders and 34 non-responders and three covariates, the logistic-regression models satisfied the conventional events-per-variable threshold of at least 10. Subgroup and regression findings from this modest sample are nonetheless reported as exploratory.

### Handling of missing data

Missing data were minimal (<5% across all variables). Analyses were performed using complete case analysis, retaining only participants with data available for all variables in a given test. Change scores and binary clinical response variables were computed according to the revision-defined analysis plan.

### Use of AI-assisted technologies

During the revision of this manuscript, the authors used AI-assisted tools (Anthropic's Claude and OpenAI's Codex, a coding assistant based on OpenAI GPT models) to support the statistical reanalysis and the drafting of text. Specifically, these tools helped write and refactor the Python analysis code (pandas, statsmodels, SciPy) that produced the reported results, assisted in drafting and editing manuscript prose during revision, and assisted in preparing the point-by-point response to reviewers. The authors evaluated the validity of all AI-assisted output: every statistical result was independently reproduced from the de-identified source data using the openly deposited analysis code (see Data Availability), and all AI-drafted or AI-edited text was reviewed, verified, and revised by the authors. The authors take full responsibility for the content of the manuscript; all hypotheses, interpretations, results, conclusions, limitations, and implications reported here represent the authors’ own ideas. No AI tool is listed as an author, and no primary research data were generated, altered, or fabricated by AI tools.

## Results

### Participant characteristics

Eighty pediatric patients undergoing preoperative procedures were enrolled between October 2023 and June 2024. The mean age of pediatric patients was 10.4 ± 3.0 years (range 6–15 years), with higher proportion of females (68.8%, 55/80) than males (31.2%, 25/80). The virtual reality intervention duration averaged 9.3 ± 4.2 minutes with median of 9.0 minutes (interquartile range 6.0-11.2 minutes, range 5–20 minutes).

Among 65 parent or caregiver participants, most were mothers (81.5%, 53/65) with remainder being fathers (18.5%, 12/65). The majority of parents (58.5%, 38/65) were between 31–40 years of age. Educational background varied, with largest group (38.5%, 25/65) having university or professional degrees. Almost two-thirds (64.6%, 42/65) reported previous emergency department visits with their child. Among 29 participating nurses, all completed satisfaction and feasibility assessments. Participant demographic characteristics are summarized in Table 1.

**Table 1:** Demographic characteristics of study participants.

Characteristics	N (%) or Mean (SD)
Study Participants (N = 80)	
Age, years (mean ± SD)	10.4 ± 3.0
Age range (years)	6–15
Gender, n (%)	
Male	25 (31.2%)
Female	55 (68.8%)
VR Duration (min)	
Mean (SD)	9.3 ± 4.2
Median (IQR)	9.0 (6.0-11.2)
Range	5-20
Parent/Caregiver Characteristics (N = 65)	
Relationship to Child, n(%)	
Mother	53(81.5%)
Father	12(18.5%)
Parent Age, n (%)	
20-30 years old	11 (16.9%)
31-40 years old	38 (58.5%)
41-50 years old	10 (15.4%)
51-60 years old	6 (9.2%)
Parent Education, n (%)	
University/ Professional Degree	25 (38.5%)
Diploma/Certificate	18 (27.7%)
High school	10 (15.4%)
Some Post Secondary University	10 (15.4%)
Elementary school	2 (3.1%)
Prior ED visit	
Yes	42 (64.6%)
No	23 (35.4%)

Abbreviations: ED = Emergency Department; IQR = Interquartile Range; SD = Standard Deviation; VR = Virtual Reality.

### Effect of virtual reality on distress and fear

Both self-reported distress and fear decreased significantly between the pre-VR (T1) and immediately post-VR (T2) assessments. Median distress on the 0–10 Numerical Rating Scale fell from 3.0 (interquartile range 2.0-5.0) to 1.0 (0.0-2.25), a median within-subject reduction of 2.0 points (Wilcoxon signed-rank test, p < 0.001; Table 2). Median fear on the 0–4 Children's Fear Scale fell from 1.0 to 0.0, a median reduction of 1.0 point (p < 0.001; Table 2). Under the primary response definitions, 46 of 80 children (57.5%) achieved a clinically meaningful distress reduction (a reduction of at least 2 points) and 44 of 80 (55.0%) a clinically meaningful fear reduction (a reduction of at least 1 point); the percentage-based secondary thresholds are reported in Table 3. Because the initiative included no concurrent comparison group, these are within-subject changes and cannot be attributed to virtual reality alone.

**Table 2:** Pre-post comparison of psychological outcome measures.

Outcome Measure	Pre-VR Median [IQR]	Post-VR Median [IQR]	Median Change (95% CI)	P-value*
Distress Score (0–10)	3.0[2.0–5.0]	1.0 [0.0–2.2]	- 2.0 (-2.50, -1.50)	<0.001
Fear Score (0–4)	1.0[0.0–2.0]	0.0 [0.0–1.0]	-1.0 (-1.00, -0.50)	<0.001

*Wilcoxon signed-rank test for non-parametric paired comparisons. Abbreviations: CI, confidence interval; IQR, interquartile range; VR, virtual reality.

**Table 3:** Clinical significance achievement by outcome.

Outcome	Patients Achieving Threshold n/n (%)	95% CI
Distress — primary: ≥ 2-point reduction	46/80 (57.5)	46.6 - 67.7
Fear — primary: ≥ 1-point reduction	44/80 (55.0)	44.1 - 65.4
Distress — secondary: ≥ 30% reduction	53/80 (66.2)	55.4 - 75.7
Distress — secondary: ≥ 50% reduction	46/80 (57.5)	46.6 - 67.7

*Abbreviations: CI = confidence interval. Primary thresholds are the revision-defined absolute changes (distress ≥2 points on the 0–10 scale; fear ≥1 point on the 0–4 scale); percentage-based thresholds are reported as labeled secondary analyses. Percentage thresholds are not applied to the 0–4 fear scale because they are baseline-dependent and undefined for the 22 of 80 participants with a baseline fear score of 0. Confidence intervals are Wilson score intervals.*

### Physiological outcomes

Analysis of physiological parameters revealed significant within-subject reductions between the pre- and post-VR assessments. Mean heart rate decreased from 86.3 ± 14.7 beats per minute pre-intervention to 80.5 ± 12.7 beats per minute post-intervention, resulting in mean reduction of 5.8 ± 13.0 beats per minute. This decrease was statistically significant (paired t-test t = 3.95, p < 0.001). Similarly, systolic blood pressure demonstrated significant reduction from baseline 113.9 ± 10.6 mmHg to post-intervention 109.8 ± 10.9 mmHg, with mean decrease of 4.1 ± 8.5 mmHg (paired t-test t = 4.31, p < 0.0001). Changes in physiological markers are presented in Table 4. Because outcomes were measured without a concurrent control group, these pre-to-post changes describe within-subject change and are not adjusted for natural settling, acclimation, or non-specific attention.

**Table 4:** Changes in physiological markers following VR intervention.

Variable	Pre-VR	Post-VR	Mean Change (95% CI)	P value*	t-statistic
Heart Rate (bpm)					
Mean ± SD	86.3 ± 14.7	80.5 ± 12.7	-5.8 (-8.65, -2.85)	<0.001	3.95
Systolic Blood Pressure (mmHg)					
Mean ± SD	113.9 ± 10.6	109.8 ± 10.9	-4.1 (-5.98, -2.20)	<0.0001	4.31

Footnote: P-values were calculated using a paired t-test. Abbreviations: BP, blood pressure; bpm, beats per minute; SD, standard deviation.

### Predictors of clinical response

Two separate logistic regression models examined predictors of clinically significant reduction (≥50% improvement) in fear and distress. The model for fear reduction was statistically significant overall (log-likelihood ratio p-value = 0.038, pseudo R^2^ = 0.077). Baseline fear score emerged as the only statistically significant predictor of substantial fear reduction (odds ratio 1.80, 95% confidence interval 1.11-2.91, p = 0.017), indicating that for each one-point increase in baseline fear score, the odds of achieving clinically significant reduction increased by 80%. Neither age (odds ratio 0.89, 95% confidence interval 0.75-1.05, p = 0.162) nor virtual reality duration (odds ratio 0.99, 95% confidence interval 0.88-1.11, p = 0.832) demonstrated significant associations with likelihood of fear reduction.

The model for distress reduction was statistically significant (log-likelihood ratio p-value = 0.040, pseudo R^2^ = 0.076). Baseline distress score was identified as significant predictor (odds ratio 1.31, 95% confidence interval 1.06-1.63, p = 0.014), indicating that each one-point increase in baseline distress was associated with a 31% increase in odds of achieving clinically significant reduction. As with the fear model, neither age (odds ratio 0.92, 95% confidence interval 0.78-1.09, p = 0.362) nor virtual reality duration (odds ratio 0.99, 95% confidence interval 0.88-1.11, p = 0.829) significantly predicted distress reduction. Predictor analyses are presented in Table 5.

**Table 5:** Logistic regression analysis: Predictors of clinical response to VR intervention.

A. Predictors of Fear Reduction (≥50% improvement)					
Variable	β Coefficient	SE	OR (95% CI)	P-value	Model Statistics
Intercept	-0.16	1.17	-	0.890	
Age (years)	-0.12	0.09	0.89 (0.75, 1.05)	0.162	
Baseline Fear (points)	0.59	0.25	1.80 (1.11, 2.91)	*0.017	Pseudo R^2^ = 0.077
VR Duration (min)	-0.01	0.06	0.99 (0.88, 1.11)	0.832	LLR p-value: 0.038
**B. Predictors of Distress Reduction (≥50% improvement)**					
**Variable**	**β Coefficient**	**SE**	**OR (95% CI)**	**P-value**	**Model Statistics**
Intercept	0.32	1.22	-	0.789	
Age (years)	-0.076	0.08	0.92 (0.78, 1.09)	0.362	
Baseline Distress (points)	0.273	0.11	1.31 (1.06, 1.63)	*0.014	Pseudo R² = 0.076
VR Duration (min)	-0.012	0.06	0.99 (0.88, 1.11)	0.829	LLR p-value: 0.040

Abbreviations: CI = confidence interval; LLR = log-likelihood ratio; OR = odds ratio; SE = standard error. Model fit: N = 80; Responders = 46; Non-responders = 34; Predictors = 3; EPV(min)=11.3; Pseudo R² = 0.076; LLR test p = 0.040; Converged = Yes. Notes: Outcome = indicator of ≥50% reduction in distress from baseline to post-VR. Odds ratios from logistic regression by ordinary maximum likelihood. OR>1 indicates higher odds of meeting the responder threshold per 1-unit increase in the predictor. These predictor models retain the secondary percentage-based responder definition (≥50% reduction) rather than the absolute primary definition used in Table 3, and model A was fitted on the fear scale as originally recorded (1–5); see Statistical Analysis. This is a legacy exploratory predictor analysis; it is not part of the primary clinical-response threshold framework and its responder definitions are not directly comparable with those in Table 3. The summary model-fit statistics above (Responders = 46; Non-responders = 34; EPV(min)=11.3; Pseudo R² = 0.076; LLR p = 0.040) describe model B (distress); the corresponding values for model A (fear) are Responders = 36; Non-responders = 44; EPV(min)=12.0; Pseudo R² = 0.077; LLR p = 0.038.

### Stakeholder experience

#### Parent and caregiver outcomes.

Analysis of parent and caregiver outcomes (n = 65) revealed consistently positive responses to virtual reality intervention. Parent satisfaction with procedure was high, with mean score of 4.55 ± 0.69 on 5-point scale (95% confidence interval 4.38-4.72), indicating strong approval of overall procedural experience. Satisfaction with anxiety management was rated at 4.18 ± 0.90 (95% confidence interval 3.96-4.40). Notably, 95.4% of parents and caregivers (62/65) indicated they would recommend virtual reality intervention for other children undergoing similar procedures (95% confidence interval 87.1-99.0%).

#### Pediatric patient outcomes.

Pediatric patients reported high levels of satisfaction with virtual reality intervention. Mean overall happiness score was 4.34 ± 0.83 on 5-point scale (95% confidence interval 4.16-4.52). Satisfaction ratings were predominantly positive, with 86.2% reporting high (score = 4) or very high (score = 5) satisfaction. Only 2.4% of participants reported low satisfaction (scores 1–2). Large majority of children (91.2%, 73/80) reported willingness to try virtual reality again in future procedures (95% confidence interval 82.8-96.4%), while 88.8% (71/80) specifically indicated that virtual reality helped with their anxiety (95% confidence interval 79.7-94.7%). Adverse effects were minimal, occurring in only 3.8% (3/80, 95% confidence interval 0.8-10.6%) of participants.

#### Healthcare provider outcomes.

Nursing staff (n = 29) reported positive responses to integrating virtual reality into pediatric procedural care. The majority of nurses (82.8%) rated the virtual reality system as “extremely easy” to manage during clinical procedures (mean implementation ease score 4.72 ± 0.53 on 5-point scale). Satisfaction among nursing staff was consistently high, with 75.9% (22/29) reporting being “very satisfied” and 17.2% (5/29) “satisfied” with intervention. No nurses reported dissatisfaction with virtual reality implementation. Notably, all participating nurses (100%, 29/29) indicated they would recommend virtual reality for pediatric procedures. Only 6.9% (2/29) of nurses reported any workflow disruption associated with intervention, while 93.1% (27/29) reported that virtual reality enhanced patient care. Stakeholder satisfaction and acceptance outcomes are presented in Table 6.

**Table 6:** Stakeholder satisfaction and acceptance outcomes.

A. Parent/Caregiver Experience (n=65)		
Outcome Measure	n (%) or Mean ±SD	95% CI
Procedure Satisfaction (1-5)	4.55 ±0.69	(4.38, 4.72)
Anxiety Management Satisfaction (1-5)	4.18 ±0.90	(3.96, 4.40)
Would Recommend VR (n, %)	62/65 (95%)	(87%, 99%)
**B. Pediatric Patient Experience (n=80)**		
Outcome Measure	n (%) or Mean ±SD	95% CI
Overall Happiness Score (1-5)	4.34 ±0.83	(4.16, 4.52)
Would Try VR Again(n, %)	73/80 (91%)	(82%, 96%)
VR Helped with Anxiety	71/80 (88 %)	(79%, 94%)
Experienced Side Effect	3/80 (3%)	(0.8%, 10%)
**C. Nursing Staff Experience (n=29)**		
**Outcome Measure**	**n (%) or Mean (SD)**	
Ease of Implementation (1-5)	4.72 ±0.53	
Overall Satisfaction (1-5)	4.69 ±0.60	
Would Recommend VR	29/29 (100%)	
Workflow Disruption	2/29 (6%)	
Enhanced Patient Care	27/29 (93%.)	

*Statistically significant reduction (p < 0.01).

### Parent anxiety outcomes

Analysis of parental anxiety revealed statistically significant reduction following virtual reality intervention (p < 0.0015). Median State-Trait Anxiety Inventory-6 score pre-intervention was 14.0 (interquartile range 11.0-17.0) and remained 14.0 post-intervention but with narrower distribution (interquartile range 11.0-15.0). Median change in anxiety score was -1.0 (interquartile range -4.00 to 0.00) with 95% confidence interval of -2.00 to 0.00.

Distribution of change scores indicated that 53.8% (35/65) of parents experienced improved anxiety levels following their child's virtual reality intervention, while 23.1% (15/65) showed no change and 23.1% (15/65) reported increased anxiety. Parent anxiety outcomes are presented in Table 7.

**Table 7:** Parent/caregiver anxiety (n = 65).

Outcome Measure	Median [IQR]	95% CI	P-value
Pre-VR Anxiety Score	14 [11–17]	[13, 15]	
Post-VR Anxiety Score	14 [11 –15]	[12, 15]	
Median Change	-1 [-4 - 0]	[-2, 0]	<0.0015

### Parent-child concordance in virtual reality acceptance

Because more than one cell of the two-by-two table had an expected count below five, the association between parent's willingness to recommend virtual reality and child's willingness to use it again was assessed with Fisher's exact test. There was no statistically significant association between the two (Fisher's exact test, p = 0.29). In 57 of 65 dyads (87.7%), parent and child responses were concordant. Most notably, 56 dyads (86.2%) demonstrated mutual positive acceptance of intervention. Discordant responses were relatively uncommon, with 2 cases (3.1%) where children were willing to use virtual reality again but parents would not recommend it, and 6 cases (9.2%) where parents would recommend virtual reality despite the child's unwillingness to use it again. Contingency analysis is presented in Table 8.

**Table 8:** Contingency table of parent and child VR acceptance.

	Child Willing to Use VR Again	
**Parent Recommends VR**	No	Yes
No	1	2
Yes	6	56

### Qualitative findings: Parent perspectives

Thematic analysis of open-ended feedback from 63 parents yielded six distinct themes regarding perceptions of virtual reality intervention. Engagement and Distraction emerged as most prevalent theme, present in 52.4% (33/63) of parent responses. This theme encompassed comments about virtual reality's capacity to capture children's attention and divert it from clinical procedures. As one parent noted, “He was distracted and didn't notice when the needle went in,” while another commented, “It takes away the attention of the child.”

Emotional Impact was identified in 34.9% (22/63) of responses, making it second most frequent theme. Parents described virtual reality intervention's effect on reducing anxiety and promoting calm for both children and parents. One parent of child with special needs observed, “He has autism and he seems much more relaxed while wearing the VR. He's not thinking about the procedure while waiting.” Another simply stated, “Very cool to watch, helpful to know my child was not in pain.”

Clinical Utility appeared in 31.7% (20/63) of parent comments, focusing on practical benefits of virtual reality in healthcare setting. Parents recognized broader applications beyond their individual experience, with comments such as “will help a lot of kids and their parents” and “It helps remove the thoughts of over thinking and worry.”

Stakeholder Experience (19.0%, 12/63) captured distinct perspectives of different individuals involved in virtual reality implementation. Some parents contrasted their own experience with their child's: “If my child chose it, sure. To me it's a bit overwhelming to have so much going on.”

Challenges and Concerns were mentioned in only 4.8% (3/63) of responses, primarily from parents reporting neutral satisfaction. These comments addressed implementation difficulties or perceived limitations: “I think it was difficult to get a vein because the skin was thick, and it hurt.”

Technology Experience was least frequent theme (3.2%, 2/63), with limited comments specifically addressing virtual reality technology itself rather than its effects: “This headset was a great idea.”

Analysis of theme co-occurrence revealed that Clinical Utility and Engagement and Distraction frequently appeared together (9 responses), suggesting parents connected virtual reality's ability to distract with its practical clinical value. Similarly, Engagement and Distraction frequently co-occurred with Stakeholder Experience (9 responses), indicating parents often framed observations of virtual reality intervention in terms of their child's engagement and their own observations. Thematic analysis summary is presented in Table 9.

**Table 9:** Thematic analysis of parent perspectives on VR intervention (N = 63).

Theme	Description	Prevalence	Representative Quotation
Engagement & Distraction	VR's capacity to capture attention, provide distraction from medical procedures, and create an engaging experience	33 (52%)	“He was distracted and didn't notice when the needle went in”
Emotional Impact	Effect on emotional states, including reduction of anxiety, fear, and promotion of calm	22 (35%)	“He has autism, and he seems much more relaxed while wearing the VR. He's not thinking about the procedure while waiting”
Clinical Utility	Practical benefits and applications in the clinical setting	20 (32%)	“Will help a lot of kids and their parents”
Stakeholder Experience	Distinct experiences and perspectives of different stakeholders	12 (19%)	“If my child chose it, sure. To me it's a bit overwhelming to have so much going on”
Challenges and Concerns	Implementation difficulties or perceived limitations	3 (5%)	“I think it was difficult to get a vein because the skin was thick, and it hurt.”
Technology Experience	Perceptions of the VR technology itself	2 (3%)	“This headset was a great idea”

### Sensitivity analyses

The robustness of the paired pre-post findings was examined through multiplicity correction (Holm and Benjamini-Hochberg; all four primary outcomes remained significant), regression-to-the-mean and analysis-of-covariance treatments of the baseline-response coupling, a bootstrap optimism-corrected assessment of the predictor models’ discrimination, and influence diagnostics. These analyses are reported in full in the Supporting Information (S2 Text) and are reproducible from the deposited code; they support the paired within-subject reductions while indicating that the baseline-as-predictor associations are largely attributable to regression to the mean rather than to differential efficacy.

## Discussion

In this single-arm initiative, immersive virtual reality intervention was associated with significant within-subject reductions in preoperative anxiety in pediatric patients, alongside high acceptance across children, parents, and healthcare providers. The findings provide preliminary, uncontrolled evidence on virtual reality in pediatric perioperative care, addressing implementation feasibility and within-subject changes in anxiety under real-world conditions; they do not establish comparative clinical effectiveness.

### Within-subject changes and possible mechanisms

The primary outcomes showed significant within-subject reductions in children's distress and fear in the preoperative holding period. Median self-reported distress scores decreased from 3 to 1 on the 0–10 scale, and fear scores dropped from 1 to 0 on the 0–4 pictorial scale, both representing highly significant improvements. In practical terms, under the revision-defined primary thresholds, 46 of 80 children (57.5%) achieved a clinically meaningful distress reduction and 44 of 80 (55.0%) a clinically meaningful fear reduction, consistent with established clinical-significance thresholds for pediatric self-report scales.

The concurrent reductions in heart rate and systolic blood pressure are consistent with reduced sympathetic arousal and provide objective physiological correlates of the self-reported reductions. In the absence of a control group, however, these changes cannot be attributed specifically to VR rather than to acclimation, natural settling, or non-specific attention. Recent pediatric virtual reality studies have documented similar physiological benefits, with investigations reporting significant reductions in electrodermal activity (68% decrease) and blood volume pulse amplitude (53% decrease) during virtual reality exposure in dental procedures, as well as comparable heart rate reductions in virtual reality versus control groups during medical procedures [10,11]. These autonomic improvements are particularly significant in pediatric populations where anxiety-induced cardiovascular activation can complicate anesthesia induction, increase procedural risk, and prolong recovery.

These objective physiological correlates add to growing evidence on virtual reality as a non-pharmacological option and motivate controlled evaluation before routine integration [12,13,15,16].

### Predictors of treatment response

The study identified factors associated with greater benefit from virtual reality intervention, with baseline anxiety levels emerging as primary predictors of clinical response. Children with higher initial distress and fear scores experienced the largest improvements. For each one-point increase in baseline fear (measured on the fear scale as originally recorded, 1–5; see Table 5), the odds of marked fear reduction were approximately doubled (odds ratio 1.8), and each additional point of baseline distress (0–10 scale) was associated with a 31% increase in the odds of major distress relief. These associations indicate that children with higher baseline anxiety showed the largest reductions (see Limitations). This pattern is consistent with regression to the mean, whereby participants with higher baseline symptom severity have greater room for measured improvement [14]. The decomposed exploratory analysis in S1 Text and S1 Table indicates that this association varies by outcome: modest for distress response, absent for willingness to use virtual reality again, and inverse for whether the child reported virtual reality helped. We do not interpret baseline severity as a consistent predictor of benefit.

Age did not emerge as significant moderator of the observed anxiety reductions across the 6–15 year range, contrasting with mixed findings in existing literature [7,13,17]. While some studies suggest younger children (4–8 years) may show greater anxiety reductions during needle procedures, other research including meta-analytic evidence found no significant age moderation of virtual reality effectiveness for pediatric anxiety during needle procedures within 4–12 year age range. Our results are consistent with anxiety reductions across the 6–15 year range, though without a comparator we cannot isolate the contribution of virtual reality's immersive features. However, because we included wide age range, it is possible that age-related differences in how children respond to intervention were not fully captured.

Regarding session duration (5–20 minutes), duration was not significantly associated with either ≥50% response outcome in the exploratory predictor models. Professional guidelines recommend 10–15 minute sessions for school-age children to optimize benefits while minimizing potential adverse effects. Notably, in earlier quality improvement cycles longer sessions (≥10 minutes) tended to yield somewhat larger anxiety reductions on average; because session duration was changed by protocol across quality improvement cycles, however, duration is confounded with cycle and these data cannot identify an optimal session length. In practice, allowing children to use virtual reality for as long as they desire within workflow constraints appears reasonable.

### Stakeholder acceptance and system integration

The comprehensive stakeholder outcomes reflect multi-level implementation success for virtual reality intervention, indicating alignment of positive experiences across all key healthcare participants. Consistently high satisfaction ratings across parents and caregivers (mean 4.55 ± 0.69), pediatric patients (mean 4.34 ± 0.83), and nursing staff (mean 4.72 ± 0.53) align with recent implementation studies showing that successful virtual reality integration requires stakeholder alignment and acceptance [18,19]. High level of parental willingness to recommend virtual reality to other families (95.4%) and children's expressed desire to use virtual reality again (91.2%) mirrors findings from other pediatric virtual reality implementation studies, where high satisfaction rates and positive user experiences drive sustained adoption and clinical integration.

Healthcare provider acceptance, with 100% of nurses recommending virtual reality and 93.1% reporting enhanced patient care, reflects critical implementation facilitators identified in multi-stakeholder research, including ease of use, minimal workflow disruption, and observed patient benefits. The significant reduction in parental anxiety (p < 0.001) with 53.8% of parents experiencing improved anxiety levels following their child's virtual reality intervention is consistent with studies suggesting that family-centered virtual reality interventions may be associated with reduced caregiver distress in pediatric healthcare settings, although the uncontrolled design precludes attributing this change to VR. Our finding that parents had significantly lower state anxiety after virtual reality session represents important but underexplored aspect of pediatric virtual reality interventions, raising the hypothesis, to be tested in controlled studies, that virtual reality may indirectly ease caregiver distress by reducing the child's anxiety.

The high degree of parent-child concordance in virtual reality acceptance (87.7% agreement) with predominantly mutual positive responses (86.2% of dyads) suggests successful alignment between patient and family preferences, critical factor in pediatric healthcare decision-making. Absence of statistical association between parent and child virtual reality acceptance (Fisher's exact test, p = 0.29) provides no evidence of an association between parent and child acceptance; given only three parent non-recommendations, this null result should not be interpreted as evidence that virtual reality is independently effective, and the high marginal acceptance in each group is described separately above, enabling implementation approaches that can work with various family situations. These findings collectively indicate that virtual reality implementation at this single site was accompanied by consistent satisfaction across participating stakeholder groups; whether that acceptability extends to other patient populations or healthcare settings was not assessed.

### Qualitative insights and implementation lessons

Qualitative analysis revealed meaningful parent perspectives highlighting impact of virtual reality interventions on pediatric healthcare experiences. The predominance of “Engagement and Distraction” as most frequently cited theme (52.4% of responses) aligns with established literature demonstrating that virtual reality's capacity to redirect children's attention away from medical procedures represents fundamental mechanism underlying its clinical effectiveness. Parent observations such as “He was distracted and didn't notice when the needle went in” support research findings that immersive virtual reality environments may engage attention in a manner consistent with attention-based distraction models.

The “Emotional Impact” theme (34.9% of responses) particularly resonated with parents of children with special needs, with one parent noting enhanced relaxation in their child with autism, reflecting virtual reality's documented benefits across diverse pediatric populations including those with neurodevelopmental conditions. The emergence of “Clinical Utility” (31.7% of responses) suggests that parents recognized virtual reality's broader healthcare applications beyond their immediate experience, indicating stakeholder appreciation for intervention's systemic value in pediatric care settings.

The minimal occurrence of “Challenges and Concerns” (4.8% of responses) mirrors implementation studies showing high acceptability rates and low adverse event profiles for pediatric virtual reality interventions. The nine-month implementation project demonstrated that even busy pediatric preoperative unit can integrate virtual reality without disrupting clinical workflow. Several critical implementation lessons emerged from systematic Plan-Do-Study-Act cycle approach. The iterative refinement process revealed that successful virtual reality integration requires flexibility in session duration, with shift from rigid 3–5 minute protocols to patient-directed 5–20 minute sessions while maintaining surgical scheduling efficiency.

The expansion from narrow procedural support to comprehensive preoperative anxiety management significantly broadened intervention's clinical utility, addressing both acute procedural fears and generalized waiting-period anxiety affecting majority of pediatric patients. Successful integration of family-centered care through tablet-based mirroring addressed parental concerns about content exposure while enhancing collaborative care experience. Gradual increase in staff comfort observed across cycles is consistent with growing staff familiarity, with nursing satisfaction and workflow integration improving over the implementation period; with a single site and no comparator we cannot characterize this as a predictable learning curve. Achievement of zero equipment failures and consistent 7.2-minute average setup times in final cycle indicates technical reliability under real-world clinical conditions over the study period; longer-term operational sustainability was not assessed.

The qualitative and quantitative findings both described engagement and perceived emotional benefits, but this exploratory single-site analysis did not identify determinants of successful or sustainable adoption.

### Study strengths and limitations

Several strengths enhance confidence in findings and distinguish this investigation from previous research in pediatric virtual reality applications. The real-world clinical implementation represents fundamental strength, as data were collected during actual preoperative care in high-volume pediatric surgical environment rather than controlled research conditions. This real-world validity ensures that findings reflect achievable outcomes under routine clinical practice, accounting for variability, time constraints, and operational complexities of hospital settings.

The iterative Plan-Do-Study-Act methodology used during quality improvement phase enabled continuous refinement and validation of intervention protocols across five systematic cycles. By the time data collection was complete, the intervention protocol had been refined and stabilized; because the intervention was not constant across cycles, however, this evolution is also an internal-validity limitation (see Limitations) rather than a source of reliability. This dynamic optimization process, rarely reported in traditional research studies, produced evidence-driven modifications such as flexible session durations and parent viewing capabilities; because these changes were confounded with quality improvement cycle, their independent contribution to later outcomes was not assessed.

Comprehensiveness of outcome measures represents another notable strength. By including psychological, physiological, and experiential measures, the study provided overall evaluation of intervention impact. Consistency of evidence across reductions in subjective fear and distress, objective physiological measures, and stakeholder satisfaction ratings reduces the likelihood that the observed within-subject changes reflect any single type of bias or measurement artifact. Integration of qualitative thematic analysis from parent feedback provides rich contextual understanding of intervention mechanisms, with themes such as “Engagement and Distraction” and “Emotional Impact” consistent with attention-based distraction frameworks.

The custom-designed virtual reality content, developed specifically for target population and clinical setting, may strengthen internal validity by linking any observed changes to the purposefully designed virtual reality experience rather than to generic technology, although the absence of a comparison group still precludes causal attribution. Technical reliability demonstrated through zero equipment failures across 80 implementations indicates that current virtual reality hardware meets clinical usage requirements, eliminating technological confounds from outcome assessment and supporting feasibility of virtual reality integration in busy clinical environments. The multidisciplinary collaborative approach involving pediatric physicians, child life specialists, nurses, and research team in both implementing and evaluating virtual reality intervention facilitated systematic refinement through quality improvement cycles while ensuring findings are credible and practical for similar pediatric healthcare settings.

Despite these contributions, several methodological limitations must be acknowledged. The single-arm, pre-post study design without control group represents most significant limitation, preventing definitive causal attribution of observed improvements solely to virtual reality intervention. Natural anxiety reduction over time, environmental acclimation, or placebo effects could potentially account for some observed changes, although magnitude and consistency of improvements combined with stakeholder observations are notable, the contribution of temporal factors and other non-specific effects cannot be excluded in this uncontrolled design. Relatedly, the finding that children with higher baseline anxiety showed the largest reductions must be interpreted in light of regression to the mean, whereby extreme initial scores tend to move toward the mean on re-measurement irrespective of any intervention [14]; because baseline values are mathematically coupled to within-subject change, the baseline-as-predictor associations reported above may be partly attributable to this artifact rather than to a differential treatment effect, and warrant confirmation using approaches that account for it (e.g., analysis of covariance or baseline-stratified analysis) in controlled studies. Observed changes may also be influenced by unmeasured confounders not controlled in this uncontrolled design, including premedication, the degree of parental presence, and procedural differences across cases. Potential for response bias constitutes another limitation, as participants’ awareness of receiving novel intervention may have generated positive expectations that influenced self-reported outcomes through demand characteristics or social desirability bias. Generalizability limitations arise from single-center academic medical center setting and sample characteristics, including predominantly educated parents with previous healthcare experience and children aged 6–15 years undergoing elective procedures. These factors may limit applicability to community hospitals, emergency surgical contexts, or populations with different technological familiarity or healthcare literacy levels. Measurement limitations include reliance on brief, simplified anxiety scales that, while practical and child-friendly, may lack sensitivity of comprehensive anxiety assessments and could contribute to ceiling or floor effects. The immediate-term outcome assessment represents another limitation, as longitudinal follow-up was not conducted to evaluate whether preoperative anxiety reduction translated to improved anesthesia induction compliance, reduced postoperative complications, or enhanced recovery outcomes. Finally, the largely positive implementation and satisfaction findings are best read as feasibility and acceptability signals rather than as evidence of effectiveness: enthusiasm for a novel technology, voluntary participation, and the absence of a comparator may each favor positive appraisal, and these results accordingly warrant confirmation in controlled implementation studies. External validity is similarly constrained: the initiative was conducted at a single pediatric preoperative holding area with a convenience sample, dedicated Child Life and nursing support, and a specific headset and content library, in children aged 6–15 years. The findings may not generalize to settings with different staffing, workflow, patient mix, or virtual reality hardware and content, and confirmation across multiple centers and implementation contexts is needed.

Finally, inability to blind participants or providers to virtual reality intervention could introduce bias in outcome reporting or patient management, although multi-stakeholder consistency of positive findings and objective physiological improvements support validity of observed effects. Despite these limitations, multi-faceted consistency of results, methodological rigor of implementation approach, and comprehensive stakeholder evaluation support further controlled evaluation of virtual reality in pediatric preoperative care while identifying clear directions for future randomized controlled investigations.

### Clinical and research implications

This study provides preliminary, uncontrolled support for the feasibility of VR as a non-pharmacological adjunct for managing pediatric preoperative anxiety; routine adoption would be premature pending controlled trials. The overall level of anxiety reduction observed represents a clinically meaningful improvement that complements existing non-pharmacological approaches. Successful integration will require institutional investment in equipment, staff training, and workflow adaptation, but our findings show that VR can be implemented in busy surgical environments without disrupting care. Iterative refinements, including flexible session durations and parent engagement strategies, enhanced both feasibility and acceptability, offering a practical framework for replication in other pediatric healthcare systems.

The exploratory analysis in S1 Table raises the possibility of patient-tailored use, though the association between baseline fear and benefit was weak and consistent with regression to the mean for several outcomes. We do not consider the current data sufficient to support preoperative screening for prioritization, and view this as a question for future controlled study. Nevertheless, given the high satisfaction and the few, minor reported adverse effects (3 of 80, 3.8%), virtual reality appears acceptable and feasible to offer in this setting, pending confirmatory controlled evaluation.

Future research should focus on rigorous randomized controlled trials comparing VR with both standard care and active non-pharmacological comparators. These studies should include economic evaluations to assess cost-effectiveness relative to pharmacological premedication and workflow efficiency. Multi-center implementation studies are also needed to test scalability across diverse healthcare environments. Additional research should explore optimization of VR content (e.g., immersive environments vs. interactive platforms), timing of delivery (anticipatory preparation vs. intraoperative distraction), and potential family-centered applications, including parent-directed VR protocols.

Finally, implementation science investigations should address practical challenges of scaling VR programs, including training models, workflow integration, and quality assurance mechanisms. Together, these directions would help establish whether VR can move from a promising innovation toward an evidence-based component of pediatric perioperative care.

## Conclusions

In this single-arm quality-improvement initiative, carefully implemented virtual reality (VR) was accompanied by meaningful within-subject improvements in the preoperative experience for children and their families, which cannot be attributed to VR alone in the absence of a comparison group. VR was associated with significant within-subject reductions in distress, fear, and physiological indicators of anxiety, while achieving high acceptance among children, parents, and healthcare providers. By evaluating psychological, physiological, and experiential outcomes, we found that VR was feasible to integrate; comparative effectiveness remains to be established.

The innovative use of custom pediatric-specific VR content, combined with an iterative quality improvement process, provides a practical framework for further controlled evaluation. Exploratory analyses raised the possibility that baseline anxiety relates to treatment benefit, though this finding is preliminary and requires confirmation in a controlled design before it can inform deployment strategy.

For healthcare systems, these results suggest VR may enhance the preoperative experience; effects on downstream outcomes such as anxiety-related complications and recovery were not measured in this initiative and remain hypotheses for future evaluation. For researchers, the work offers implementation guidance and underscores the need for rigorous trials to confirm efficacy, explore long-term outcomes, and evaluate cost-effectiveness.

With continued research and thoughtful implementation, VR is a promising candidate for inclusion in compassionate, family-centered pediatric care, pending confirmatory controlled research—helping more children approach medical procedures with confidence and calm.

## Supporting information

S1 Text Exploratory analysis of baseline anxiety as a predictor of positive outcomes. Rationale for replacing the withdrawn composite implementation-success outcome with a per-outcome exploratory analysis of baseline fear as a predictor, and interpretation of the resulting associations, including the baseline-floor coupling that inflates the fear-response odds ratio.
(DOCX)

S1 Table Baseline fear as a predictor of each component outcome (exploratory). Ordinary logistic regression of each positive outcome on baseline fear (Children's Fear Scale, 0–4), with age and virtual-reality session duration as covariates (N = 80); reports the odds ratio, 95% confidence interval, p-value, and pseudo-R-squared for each outcome.
(DOCX)

S2 Text Robustness and sensitivity analyses. Multiplicity correction of the four pre-post comparisons, regression-to-the-mean and baseline-coupling diagnostics, a bootstrap optimism-corrected assessment of the predictor models, and per-Plan-Do-Study-Act-cycle consistency checks.
(DOCX)

S2 Table Median within-subject change by Plan-Do-Study-Act cycle. Median change in distress, fear, and heart rate, together with the median virtual-reality duration, for each of the five implementation cycles.
(DOCX)
