# Supplementary material for: Virtual reality as a non-pharmacological intervention for pediatric preoperative anxiety: A quality improvement initiative and retrospective analysis
Source: PLOS Digit Health. 2026 Nov 5;5(11):e0001219. doi: 10.1371/journal.pdig.0001219 (PMC13739122; doi:10.1371/journal.pdig.0001219)
Supplement: Supplementary file 1 [file pdig.0001219.s001.docx]

*Virtual Reality as a Non-Pharmacological Intervention for Pediatric Preoperative Anxiety: A Quality Improvement Initiative and Retrospective Analysis*

**S1 Text. Exploratory analysis of baseline anxiety as a predictor of positive outcomes**

An earlier version of this work reported a single composite “implementation-success” outcome and a logistic model identifying baseline fear as its predictor. Because a single composite can obscure heterogeneous – and, for some components, statistically artifactual – associations, and because the composite’s exact construction was not reproducible, we do not report that composite. Instead, as an exploratory, hypothesis-generating analysis, we examine baseline fear (Children’s Fear Scale, 0-4) as a predictor of each positive outcome **separately**, using ordinary logistic regression with age and virtual-reality session duration as covariates (S1 Table).

**This decomposition is not a reconstruction of the original composite outcome and should not be compared numerically with the withdrawn composite model (baseline-fear OR 2.30); it is a replacement exploratory analysis using the recorded component outcomes.**

The association is **not uniform across outcomes**. Higher baseline fear is modestly associated with a greater odds of a clinically meaningful **distress** reduction, but it is **not** associated with a child’s intention to use virtual reality again and is **inversely** associated with the child’s report that virtual reality helped. The row for a clinically meaningful **fear** reduction is included only as a diagnostic: under an absolute-reduction rule, children whose baseline fear score is 0 (22 of 80) cannot register a further reduction, so baseline fear partly determines that outcome by construction. Its large odds ratio therefore demonstrates the magnitude of this baseline-floor coupling (regression to the mean; see main-text Discussion) and is **not** evidence that virtual reality works better in more fearful children. These exploratory results should be interpreted with caution and require confirmation in controlled designs. All values in S1 Table are reproduced by the deposited analysis code (NB2_revision, section S15).
