# Supplementary material for: Virtual reality as a non-pharmacological intervention for pediatric preoperative anxiety: A quality improvement initiative and retrospective analysis
Source: PLOS Digit Health. 2026 Nov 5;5(11):e0001219. doi: 10.1371/journal.pdig.0001219 (PMC13739122; doi:10.1371/journal.pdig.0001219)
Supplement: Supplementary file 2 [file pdig.0001219.s002.docx]

*Virtual Reality as a Non-Pharmacological Intervention for Pediatric Preoperative Anxiety: A Quality Improvement Initiative and Retrospective Analysis*

**S1 Table. Baseline fear as a predictor of each component outcome (exploratory)**

Ordinary logistic regression; predictor = baseline fear (CFS 0-4); covariates = age and VR session duration; N = 80. Exploratory / hypothesis-generating. Odds ratios are per one-point increase in baseline fear.

| Component outcome | n with outcome | Baseline-fear OR (95% CI) | p | Pseudo R² | Note |
| --- | --- | --- | --- | --- | --- |
| Distress clinical response (≥2-point) | 46 | 1.71 (1.05, 2.78) | 0.032 | 0.084 | – |
| Fear clinical response (≥1-point) | 44 | 7.72 (3.02, 19.74) | <0.001 | 0.338 | diagnostic only: baseline-floor coupling / regression to the mean; not a predictor finding |
| Would try VR again | 73 | 0.93 (0.37, 2.30) | 0.871 | 0.114 | – |
| VR helped with anxiety | 71 | 0.25 (0.09, 0.66) | 0.005 | 0.311 | inverse association |

*Confidence intervals and p-values are from ordinary maximum-likelihood logistic regression. These analyses are exploratory and were not pre-specified. The fear-response row is reported as a coupling diagnostic (see S1 Text), not as a component predictor result.*
