# Supplementary material for: Virtual reality as a non-pharmacological intervention for pediatric preoperative anxiety: A quality improvement initiative and retrospective analysis
Source: PLOS Digit Health. 2026 Nov 5;5(11):e0001219. doi: 10.1371/journal.pdig.0001219 (PMC13739122; doi:10.1371/journal.pdig.0001219)
Supplement: Supplementary file 3 [file pdig.0001219.s003.docx]

*Virtual Reality as a Non-Pharmacological Intervention for Pediatric Preoperative Anxiety: A Quality Improvement Initiative and Retrospective Analysis*

**S2 Text. Robustness and sensitivity analyses**

The following analyses examine the robustness of the paired pre-post findings and the interpretation of the baseline-as-predictor associations. All values are reproducible from the deposited analysis code (NB2_revision).

**Multiplicity.** Across the four pre-post outcome comparisons, all four remained significant after both Holm and Benjamini-Hochberg correction (Holm-adjusted p: distress 3.3 × 10⁻⁹; fear 6.1 × 10⁻⁶; heart rate 1.7 × 10⁻⁴; systolic blood pressure 9.4 × 10⁻⁵).

**Regression to the mean and baseline coupling.** Baseline score and within-subject change were strongly negatively correlated by construction (Spearman r = −0.71 for distress, −0.72 for fear; both p < 10⁻¹²), the signature of regression to the mean. An analysis of covariance of the post-intervention score on baseline (with age and virtual-reality duration) showed that a higher baseline score predicts a higher *post* score (distress slope 0.44; fear slope 0.37; both p < 0.001) – i.e., “more anxious” children remained relatively more anxious – rather than a better response. Consistent with this, baseline-stratified clinically-meaningful response rates rose only modestly across baseline tertiles (distress: 51%, 64%, 65% from low to high; fear: 40% low to 56% high). The large baseline-response odds ratios under the absolute-reduction rule (fear 7.72, distress 2.62) are therefore RTM/coupling artifacts and are not interpreted as evidence that virtual reality is more effective in more anxious children.

**Internal validation of the predictor models.** For the secondary (≥50%) responder models reported in Table 5, a bootstrap optimism-corrected assessment of discrimination gave modest, overfit-adjusted areas under the ROC curve (distress: apparent 0.687, optimism +0.044, corrected 0.643, events-per-variable 11.3; fear: apparent 0.704, optimism +0.040, corrected 0.664, events-per-variable 12.0). Influence diagnostics identified four high-influence observations (Cook’s distance > 4/*n*) in each model. These models are accordingly interpreted as exploratory and hypothesis-generating.

**Per-cycle consistency.** Median change by Plan-Do-Study-Act cycle is tabulated in S2 Table. A Kruskal-Wallis test detected no significant between-cycle difference in distress change (H = 3.21, p = 0.52) and a Spearman test showed no monotonic trend (rho = 0.04, p = 0.75); given the small per-cycle samples these are descriptive checks, and the non-constant intervention across cycles is retained as an internal-validity limitation.
