# Supplementary material for: Virtual reality as a non-pharmacological intervention for pediatric preoperative anxiety: A quality improvement initiative and retrospective analysis
Source: PLOS Digit Health. 2026 Nov 5;5(11):e0001219. doi: 10.1371/journal.pdig.0001219 (PMC13739122; doi:10.1371/journal.pdig.0001219)
Supplement: Supplementary file 4 [file pdig.0001219.s004.docx]

*Virtual Reality as a Non-Pharmacological Intervention for Pediatric Preoperative Anxiety: A Quality Improvement Initiative and Retrospective Analysis*

**S2 Table. Median within-subject change by Plan-Do-Study-Act cycle**

| Cycle | n | Distress change | Fear change | Heart-rate change | Median VR duration (min) |
| --- | --- | --- | --- | --- | --- |
| 1 | 10 | −1.5 | −1.0 | 0.0 | 5.0 |
| 2 | 11 | −2.0 | 0.0 | −5.0 | 6.0 |
| 3 | 13 | −1.0 | 0.0 | −4.0 | 7.0 |
| 4 | 29 | −2.0 | −1.0 | −9.0 | 10.0 |
| 5 | 17 | −1.0 | −1.0 | −7.0 | 11.0 |

*Negative values denote improvement (reduction). Distress on the 0-10 Numerical Rating Scale; fear on the 0-4 Children’s Fear Scale; heart rate in beats/min. Reproduced by NB2_revision (sections S2, S6-S8, S11, S12).*
